# Effect of Potassium Iodid on Luetin Reaction

**Published:** 1916-03

**Authors:** 


**Effect of Potassium Iodid on Luetin Reaction.** John W. Sherrick, Ann Arbor, Jour. Mich. State, Med. Soc., Jan. A positive pustular or nodular luetin reaction can be obtained in 99% of all cases, irrespective of the presence of syphilis, if KI has been administered simultaneously or shortly before the intradermal test. Agar, starch, etc., introduced intradermally instead of luetin, will give a similar reaction, but the KI must have been very recently administered. Other drugs containing iodine have the same influence.

